# Which disciplines form digital public health, and how do they relate to each other?

**DOI:** 10.1093/eurpub/ckac129.230

**Published:** 2022-10-25

**Authors:** C Pan

**Affiliations:** 1 Leibniz Institute for Prevention Research and, Epidemiology - BIPS, Bremen, Germany; 2 Leibniz ScienceCampus Digital Public Health, Bremen, Germany

## Abstract

A standard public health definition, coined by Acheson in 1998 and adapted by the World Health Organization, describes public health as ‘the art and science of preventing disease, prolonging life and promoting health through the organised efforts of society’. Several other definitions emphasise different foci such as living conditions, the efficient use and equitable distribution of resources, or sustainability. Yet, they all share the dual nature as a science and practice of public health, its focus on the health of entire populations, and its interdisciplinary nature. In particular, joint efforts of individual disciplines are needed to combine subject matter knowledge and approaches to protect and promote population health. Traditional core public health disciplines comprise the social sciences, humanities, natural sciences, and environmental sciences. Their key tasks are summarised in the ten essential public health services, which can be further extended to digital public health. This extension leads to increasing use and integration of technological innovation and advancement into public health functions which require intensive collaborations with disciplines from the engineering field. In this presentation, we aim to describe the transition from public health to digital public health, emphasising the disciplines needed to tackle population health challenges in a digitalised world. In a first step, we will illustrate core disciplines and their sub-disciplines that are traditionally known in the public health field. In a second step, we will introduce further core and sub-disciplines prominent in digital public health. Finally, we will briefly present examples of key strengths and challenges of some of the disciplines. After this presentation, workshop participants should have a first understanding of the role and importance of interdisciplinarity in digital public health. Stefanie Do will host the table on epidemiology during the world coffee.

